# Brain inflammation and hypertension: the chicken or the egg?

**DOI:** 10.1186/s12974-015-0306-8

**Published:** 2015-05-03

**Authors:** Pawel J Winklewski, Marek Radkowski, Magdalena Wszedybyl-Winklewska, Urszula Demkow

**Affiliations:** Institute of Human Physiology, Medical University of Gdansk, Tuwima Str. 15, 80-210 Gdansk, Poland; Department of Immunopathology of Infectious and Parasitic Diseases, Medical University of Warsaw, Pawinskiego Str. 3c, 02-106 Warsaw, Poland; Department of Laboratory Diagnostics and Clinical Immunology of Developmental Age, Medical University of Warsaw, Marszalkowska Str. 24, 00-576 Warsaw, Poland

**Keywords:** Brain inflammation, Neurogenic hypertension, Angiotensin II, Prorenin, Reactive oxygen species, Chronic inflammation, Obesity

## Abstract

Inflammation of forebrain and hindbrain nuclei controlling the sympathetic nervous system (SNS) outflow from the brain to the periphery represents an emerging concept of the pathogenesis of neurogenic hypertension. Angiotensin II (Ang-II) and prorenin were shown to increase production of reactive oxygen species and pro-inflammatory cytokines (interleukin-1 beta (IL-1β), interleukin-6 (IL-6), tumor necrosis factor-alpha (TNF-α)) while simultaneously decreasing production of interleukin-10 (IL-10) in the paraventricular nucleus of the hypothalamus and the rostral ventral lateral medulla. Peripheral chronic inflammation and Ang-II activity seem to share a common central mechanism contributing to an increase in sympathetic neurogenic vasomotor tone and entailing neurogenic hypertension. Both hypertension and obesity facilitate the penetration of peripheral immune cells in the brain parenchyma. We suggest that renin-angiotensin-driven hypertension encompasses feedback and feedforward mechanisms in the development of neurogenic hypertension while low-intensity, chronic peripheral inflammation of any origin may serve as a model of a feedforward mechanism in this condition.

## Introduction

Hypertension is an epidemic health challenge, a proven major risk factor of the development of cardiovascular disease and the leading cause of morbidity and mortality worldwide [1]. Despite the availability of several classes of antihypertensive drugs, the treatment of hypertension often remains suboptimal. In addition, the prevalence of uncontrolled hypertension continues to rise globally [2].

It is well established that inflammation is involved in the pathogenesis of hypertension [3]. In animals with experimental hypertension, T cells and macrophages accumulate in the kidneys and peripheral vasculature and most likely contribute to the *end organ* damage associated with this disease [4]. Mice lacking lymphocytes (Rag1-/- mice) develop blunted hypertension and are protected from vascular dysfunction and vascular oxidative stress in response to various stimuli, including angiotensin II (Ang-II), norepinephrine (NE) and deoxycorticosterone acetate plus sodium chloride (DOCA-salt). The adoptive transfer of T cells, but not B cells, restores hypertension in these animals [5]. Dentritic cells from hypertensive mice exhibit increased expression of the B7 ligands CD80 and CD86 indicating dendritic cell maturation and activation. The blocking of these co-stimulatory molecules prevents hypertension and T cell activation during both Ang-II- and DOCA-salt-induced hypertension [6].

It is generally accepted that uncontrolled, resistant hypertension is primarily of neurogenic origin - driven by chronic hyperactivity of the sympathetic nervous system (SNS) [7-9]. Clearly, the SNS stimulation of the heart, vasculature and kidneys increases blood pressure (BP) by augmenting cardiac output, vascular resistance and fluid retention [8]. However, the SNS also acts as an integrative interface between the brain and the immune system [10-12]. Primary (bone marrow and thymus) and secondary (spleen and lymph nodes) lymphoid organs are abundantly innervated by autonomic, mostly sympathetic efferent fibres. NE, the SNS primary neurotransmitter, is released into the lymphoid tissue and modulates the function of immune cells. Most of them express receptors for glucocorticoids, the end product of the hypothalamic pituitary adrenal axis. Thus, the SNS and hypothalamic pituitary adrenal axis can regulate the magnitude of innate and adaptive immune responses in multiple ways [12-14]. Additionally, adipose tissue is directly innervated by the SNS and this innervation plays an important role in metabolic and endocrine function [15]. Interestingly, perivascular adipose tissue components can release NE independently from the SNS, possibly exerting co-stimulatory local control of arterial function [16].

The SNS efferent transmission from the brain to the peripheral tissues is controlled by several forebrain and hindbrain nuclei. Particularly important are the paraventricular nucleus of the hypothalamus (PVN), the circumventricular organs (CVOs), the rostral ventral lateral medulla (RVLM), the anteroventral third ventricle (AV3V) and the nucleus of the solitary tract (NTS) [17,18]. All of these structures demonstrate high expression of Ang-II type 1 receptors (AT1R) [19]. In mammals, CVOs include the median eminence and adjacent neurohypophysis, the organum vasculosum lamina terminalis (OVLT), the subfornical organ (SFO) and the area postrema (AP). CVOs are characterized by their small size, high permeability and fenestrated capillaries, which enable polypeptide hypothalamic hormones to leave the brain without disrupting the blood-brain barrier (BBB) and permit substances that do not cross the BBB to trigger changes in the brain function (that is Ang-II or cytokines) [20].

Pro-inflammatory cytokines produced in the periphery can feed back to the brain, passing through the BBB at points of increased permeability in CVOs and/or disrupted BBB. PVN stimulation with tumor necrosis factor-alpha (TNF-α) and interleukin-1 beta (IL-1β) results in augmented adrenocorticotropic hormone release, increased sympathetic outflow and enhanced cardiac sympathetic afferent reflex with subsequent BP elevation [21-23]. However, pro-inflammatory cytokines are also produced by glia and neurons and the recently identified brain inflammatory response to peripheral inflammation may further contribute to the development of hypertension [11,24-26]. Interestingly, circulating Ang-II can signal NTS neuronal networks across the BBB [27] and contributes to BBB disruption [28-30], enabling its own access to brain areas that are normally protected by the BBB, including the PVN, RVLM, and NTS [31]. Also, pro-inflammatory cytokines (IL-1β, interleukin-6 (IL-6) and TNF-α) may cause dysregulation of adherens and tight junctions leading to BBB permeabilisation [32,33]. Therefore, the question of whether the feedback or feedforward mechanism prevails and consequently whether neuroinflammation is a cause or rather the effect of hypertension remains open.

### Renin-Ang-II system and neuroinflammation

Ang-II acting via its AT1R within the PVN is a major contributor to chronic sympathoexcitation [34]. A slow-pressor Ang-II model of hypertension has been validated in mice and rats, and it mimics the essential hypertension in humans as reviewed by Reckelhoff and Romero [35]. Anti-inflammatory cytokine interleukin-10 (IL-10) or minocycline, a tetracycline antibiotic, inhibits the activation of microglia and reduces BP in this model [36]. Furthermore, minocycline treatment eradicates the Ang-II-induced increase in mRNAs for pro-inflammatory cytokines (IL-1β; IL-6; TNF-α) and the decrease in IL-10 mRNA [36-38]. Ang-II-induced hypertension is dependent upon the activation of the inflammatory nuclear factor kappa B (NFkB) in the PVN [39], and direct injection of IL-1β into the PVN or via the intracerebroventricular (ICV) route increases mean BP [23,38,39]. Interestingly, low-intensity exercise normalizes nicotinamide adenine dinucleotide phosphate-oxidase (NADPH oxidase) and reactive oxygen species (ROS) content, suppresses TNF-α and IL-6 mRNA expression within the PVN and improves baroreflex control of the heart rate, decreases BP and reduces the low-frequency component of BP variability [40].

Protracted infusion of Ang-II using osmotic mini-pumps in mice at the rate of 400 ng/kg/min is a slow-pressor dose which raises the systolic BP by 20 to 40 mm Hg [28]. In such a model, a significant increase in leukocyte adhesion on brain venules is observed on day 4 and this effect is maintained on day 14 of Ang-II infusion. Additionally, since the day 14, the leucocyte adhesion is further accompanied by a significant increase in leukocyte rolling [41]. Importantly, BP increases on day 6 in this model [41], while oxidative stress peaks on day 4 [42] of Ang-II infusion. Therefore, it is likely that the increased leukocyte adhesion precedes the onset of hypertension but coincides with the development of oxidative stress. Furthermore, increased leukocyte adhesion in Ang-II-infused mice is associated with increased BBB permeability while the reversal of cerebrovascular inflammation (leukocyte rolling and adhesion) through tempol (superoxide dismutase mimetic) treatment is also associated with restoration of the BBB function [28]. It was observed that Ang-II affects BBB permeability in the tissue culture environment [29,30]. Importantly, the increased BBB permeability facilitates leakage of circulating Ang-II into PVN, NTS and RVLM [31].

In addition to the Ang-II/AT1R mechanism, it has been proven that another member of the renin-angiotensin system (RAS), prorenin, and the prorenin receptor (PRR) plays a role in the central control of neurogenic hypertension [43]. Prorenin, like Ang-II, increases pro-inflammatory cytokine expression (TNF-α and IL-1β) in the NTS via the NFkB complex [39,44]. Furthermore, prorenin can elicit a stronger response in cytokine production in Ang-II-pretreated microglia than in non-pretreated microglia. Prorenin-induced increases in cytokine expression are eradicated by the microglial activation inhibitor minocycline [45]. Down-regulation of PRR in the supraoptic nucleus attenuates BP development in spontaneously hypertensive rats (SHR) [46], another animal model of human hypertension [47,48]. PRR levels are higher in the PVN of human renin-angiotensinogen double transgenic hypertensive mice. Knockdown of PRR in the brains of these animals significantly decreases BP and sympathetic vasomotor tone [49]. Neuron-specific knockdown of PRR also can reduce Ang-II formation and BP in the DOCA-salt mouse model of hypertension [50,51]. Down-regulation of PRR mediated by viral transfection in the supraoptic nucleus decreases BP and the expression of inflammatory mediators [46].

The inflammatory mechanisms associated with neurogenic hypertension are not restricted to the PVN in the brain as studies indicate that the NTS exhibits an inflammatory state in SHR [52]. The theory of vascular inflammation of the brainstem has been presented elsewhere [52] and is not discussed in the current review.

SNS activity and hypertensive responses are associated with the levels of cytokines and oxidative stress markers in the RVLM. Chronic inhibition of the angiotensin-converting enzyme in the PVN attenuates both sympathoexcitation and ROS and modulates expression of cytokines (decreasing TNF-α, IL-1β, IL-6 and MCP-1 while increasing IL-10) in the RVLM in renovascular hypertension [51]. This effect is due to the high number of PVN neurons projecting to the RVLM (PVN-RVLM neurons).The number of PVN-RVLM neurons is as much as seven times the number of PVN neurons projecting to the spinal cord [53,54], and the PVN-RVLM pathway contributes to the change in SNS activity observed after activation of the PVN. Interestingly, the infusion of tempol (superoxide dismutase mimetic) in the PVN not only reduces the ROS response but also decreases BP and SNS activity in hypertension [55].

In conclusion, Ang-II and prorenin increase the production of ROS and mRNAs for pro-inflammatory cytokines (IL-1β, IL-6, TNF-α), whereas they decrease expression of IL-10 mRNA in the PVN and RVLM [36-38]. Both prorenin and Ang-II act via the NFkB complex [39,44]. Ang-II also augments BBB permeability in the tissue culture environment and Ang-II-induced hypertension [28-30]. Inflammatory response and ROS formation in the PVN and RVLM results in elevated SNS activity and hypertension. It seems that RAS-induced hypertension represents a combination of feedforward (direct Ang-II and prorenin effects on the PVN and RVLM) and feedback (systemic Ang-II and cytokines signalling via CVOs) mechanisms that may ultimately promote the development of the neurogenic form of hypertension.

### Lipopolysaccharide-induced neuroinflammation

Lipopolysaccharide (LPS), both in microglial culture and after ICV injection, elicits an inflammatory response via Toll-like receptor 4 [56,57]. An early and essential step in this process is the NADPH oxidase-dependent production of ROS, which in turn stimulates mitogen-activated protein kinase (MAPK) pathways. While LPS activates all three of the major MAPK pathways, the p38 MAPK pathway seems to be most closely associated with LPS-induced up-regulation of inflammatory mediators [58], including TNF-α, IL-1β and cyclooxygenase 2 (COX-2), the inducible form of cyclooxygenase regulating synthesis of prostaglandin E2 (PGE2) [56,57,59,60]. COX-2 and PGE2 are known to activate the SNS [21,22,61]. ICV LPS also stimulates the hypothalamic expression of AT1R mRNA, but not the angiotensin-converting enzyme mRNA, via an NADPH oxidase-dependent pathway that does not require p38 MAPK [60]. Therefore, Zhang *et al*.’s [60] study suggests that Ang-II and the pro-inflammatory cytokines may share a common mechanism for up-regulation of the AT1R. Ang-II activates NADPH oxidase-dependent superoxide production [62] and MAPK signalling pathways [63-66]. NADPH oxidase-dependent superoxide production up-regulates AT1R, which contributes to the increased sympathetic drive [67]. NADPH oxidase-dependent superoxide generation can be reduced either by blocking NADPH oxidase activity [62] or by destroying ROS [62,68]. The downstream effects of Ang-II- and LPS ROS-generating systems seem to be differentially regulated as the p38 inhibitor has no effect on LPS-induced AT1R activity but substantially reduces mRNA for TNF-α and COX-2 [60].

Intraperitoneal LPS infusion is a well-characterized rodent model of systemic inflammation. In contrast to high-dose endotoxin triggering a robust yet transient inflammatory response, low-dose endotoxin causes low-grade persistent inflammatory reactions from the host. This reaction is comparable to low-grade systemic inflammation elicited by periodontal bacteria or a hyperlipidic diet [69-72]. LPS-induced chronic systemic inflammation augments levels of TNF-α, IL-1β, IL-6 and inducible nitric oxide synthase (iNOS) in the RVLM. The expression of pro-inflammatory factors in this study [25] was comparable to that found in the brain of other animal models of hypertension with neurogenic components, including chronic infusion of Ang-II and SHR [24,73]. Oxidative stress resulting from inflammation in the RVLM is downstream to microglial activation via a COX-2-dependent mechanism. COX-2 augments expression of the pro-inflammatory cytokines and iNOS in the RVLM. The increase of protein expression and enzyme activity of COX-2 is reversible by inhibiting microglial activation. Interestingly, COX-2 activation seems to be downstream to the loss of endothelial integrity in the RVLM [25]. Microglial activation and the presence of COX-2 and cytokines ameliorate redox-sensitive expression of the Kv4.3 channel protein [25]. Kv4.3 contributes to the transient outward potassium current, and its activation diminishes neuronal excitability by increasing the duration of action potential [74]. By reducing Kv4.3 channel protein expression, neuroinflammation in the RVLM promotes hypertension via SNS activation that may result from a redox-sensitive increase in neuronal excitability. It is unlikely that the observed neuroinflammation in the RVLM resulted from direct entry of blood-borne inflammatory cytokines as the LPS-promoted long-term pressor response and the reduction in expression of the voltage-gated potassium channel Kv4.3 in the RVLM were antagonized by minocycline (an inhibitor of microglial activation), the COX-2 inhibitor, pentoxifylline (a cytokine synthesis inhibitor) or tempol (superoxide dismutase mimetic), either infused into the cisterna magna or microinjected bilaterally into the RVLM. The same treatments were ineffective against LPS-induced systemic inflammation [25].

To summarize, LPS in microglial culture or provided either centrally via ICV injection or peripherally via intraperitoneal infusion causes endothelial dysfunction and activates microglia in the brain nuclei controlling SNS efferent transmission to induce COX-2-dependent neuroinflammation and a subsequent increase in ROS production. LPS and Ang-II seem to share a common mechanism contributing to an increase in sympathetic neurogenic vasomotor tone and neurogenic hypertension [25,56,57,59,60]. Therefore, peripheral chronic inflammation may potentially lead to neurogenic hypertension without a pre-existing hypertensive condition, suggesting a feedforward brain-originated mechanism of peripheral cardiovascular pressor responses.

### Obesity-induced neuroinflammation

Obesity is proven to be a mild inflammatory condition often accompanied by increased RAS activity [26,75]. A high-fat diet (HFD) and obesity are associated with the activation of microglia [76,77] and astrocytes [76,78,79], suggesting an inflammatory state in the CNS. In rodents, HFD feeding also initiates an inflammatory cascade in both the PVN and the SFO, two brain regions that are critical for the regulation of BP and energy balance. An increased number of glial cells are Ang-II dependent, and they are localized specifically within the parvocellular portion of the PVN and the SFO [80]. Diet-induced obesity and leptin increase AT1aR expression in the SFO [80,81]. The silencing of AT1aR in mice or the pharmacological blocking of brain AT1R in rats attenuates leptin-induced SNS activity in renal and brown adipose tissue, while not attenuating leptin-induced decreases in food intake or body weight. ICV administration of captopril reverses the effects of leptin on renal and brown adipose tissue SNS activity in rats, suggesting that the production of Ang-II within the brain contributes to the interaction between the brain RAS and leptin [81]. There is a mounting evidence for a distributed brain network of leptin action, encompassing the NTS [82-84], the SFO [85,86] and the ventromedial and dorsomedial hypothalamic nuclei [87], all of which are involved in neurohumoral control of the circulation and Ang-II action.

In the normal condition, microglia exist primarily in a resting state with low levels of CD45 expression, which increases to high levels upon activation by inflammatory stimuli such as LPS and in various neurologic conditions such as Alzheimer’s disease [88-90]. For this reason, it is not possible to differentiate activated microglia and bone marrow-derived cells in the CNS only on the basis of CD45 expression. Such differentiation is feasible using the radiation bone marrow chimaera model [91]. Using this model, Buckman *et al*. showed that less than 10% of the CD11b^+^CD45^hi^ cells were of microglial origin; 93% of the bone marrow-derived cells in the CNS were found in the parenchyma but were not associated with vessels and had a distinct stellate morphology characterized by enlarged somata [92]. The hypothesis that bone marrow-derived monocytes act like macrophages in the CNS was supported by Simard *et al*. [93], who demonstrated that bone marrow-derived monocytes/macrophages enter the CNS and phagocytose and degrade amyloid more effectively than resident microglia in a mouse model of Alzheimer’s disease. The physiological significance of immune cell recruitment to the CNS during obesity and whether these cells are recruited as a consequence of obesity-induced neuroinflammation and/or contribute to the neuropathology associated with obesity have yet to be determined. It is not known if forebrain and hindbrain nuclei controlling the SNS outflow from the brain to the peripheral tissues are affected by bone marrow-derived monocytes. Importantly, the number of CNS-infiltrating monocytes is positively correlated with body weight, fat mass and markers of inflammation in adipose tissue (CD68 and CCL2 gene expression) [92].

In conclusion, obesity initiates an inflammatory cascade in both the PVN and the SFO. The increase in the number of glial cells is Ang-II dependent [80].The inflammatory ability of resident microglial cells might potentially be enhanced by HFD-induced recruitment of bone marrow-derived monocytes to the brain [92]. Therefore, obesity primarily not associated with hypertension may trigger feedforward launching of neurogenic hypertension by a brain-originated mechanism.

### Future directions

**Figure 1:**
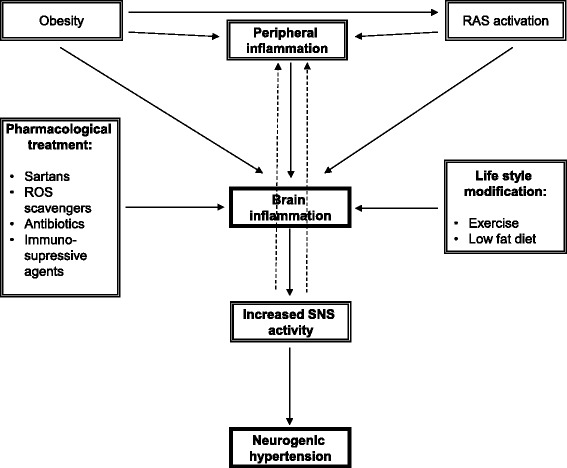
Feedforward mechanism induced by peripheral chronic inflammation leading to the development of neurogenic hypertension.

Inflammation of forebrain and hindbrain nuclei controlling the SNS efferent transmission from the brain to the periphery seems to constitute a key element in the development of neurogenic hypertension. Marvar *et al*. [49] proposed for the first time a feedforward mechanism with a central role of Ang-II in brain inflammatory response and subsequent SNS activation. We suggest that RAS-driven hypertension encompasses feedback and feedforward mechanisms, while chronic low-intensity peripheral inflammation of any origin may serve as an example of a feedforward mechanism in the development of neurogenic hypertension (Figure 1). Chronic systemic inflammation and Ang-II (or even prorenin) may share the same signalling pathways, ultimately leading to increased production of pro-inflammatory cytokines in brain nuclei controlling cardiovascular function.

Clearly, the results from animal studies discussed above open up enormous potential for translation, both in terms of pharmacological therapy and lifestyle modifications. Sartans (AT1R blockers) are already commonly used for therapy in hypertension, diabetes and stroke but have not been studied in humans in the context of their central anti-inflammatory action. Nevertheless, the safety profile of this group of drugs is relatively well known. Antibiotics, ROS scavengers and immunosuppressive agents are also likely candidates for controlled randomized trials in humans. Finally, low-intensity but repetitive exercise and a low-fat diet represent a safe approach to reducing the risk of brain inflammation and the subsequent development of neurogenic hypertension.

## References

[1] Kearney PM, Whelton M, Reynolds K, Muntner P, Whelton PK, He J (2005). Global burden of hypertension: analysis of worldwide data. Lancet..

[2] Chobanian AV (2009). Shattuck Lecture. The hypertension paradox–more uncontrolled disease despite improved therapy. N Engl J Med.

[3] Coffman TM (2011). Under pressure: the search for the essential mechanisms of hypertension. Nat Med..

[4] Muller DN, Mervaala EM, Schmidt F, Park JK, Dechend R, Genersch E (2000). Effect of bosentan on NF-kappaB, inflammation, and tissue factor in angiotensin II-induced end-organ damage. Hypertension..

[5] Guzik TJ, Hoch NE, Brown KA, McCann LA, Rahman A, Dikalov S (2007). Role of the T cell in the genesis of angiotensin II induced hypertension and vascular dysfunction. J Exp Med..

[6] Vinh A, Chen W, Blinder Y, Weiss D, Taylor WR, Goronzy JJ (2010). Inhibition and genetic ablation of the B7/CD28 T-cell costimulation axis prevents experimental hypertension. Circulation..

[7] Dampney RA, Horiuchi J, Killinger S, Sheriff MJ, Tan PS, McDowall LM (2005). Long-term regulation of arterial blood pressure by hypothalamic nuclei: some critical questions. Clin Exp Pharmacol Physiol..

[8] Esler M (2011). The sympathetic nervous system through the ages: from Thomas Willis to resistant hypertension. Exp Physiol..

[9] Takahashi H (2012). Upregulation of the renin-angiotensin-aldosterone-ouabain system in the brain is the core mechanism in the genesis of all types of hypertension. Int J Hypertens..

[10] Elenkov IJ, Wilder RL, Chrousos GP, Vizi ES (2000). The sympathetic nerve–an integrative interface between two supersystems: the brain and the immune system. Pharmacol Rev..

[11] Paton JF, Waki H (2009). Is neurogenic hypertension related to vascular inflammation of the brainstem?. Neurosci Biobehav Rev..

[12] Winklewski PJ, Radkowski M, Demkow U (2014). Cross-talk between the inflammatory response, sympathetic activation and pulmonary infection in the ischemic stroke. J Neuroinflammation..

[13] Bellinger DL, Millar BA, Perez S, Carter J, Wood C, ThyagaRajan S (2008). Sympathetic modulation of immunity: relevance to disease. Cell Immunol..

[14] Nance DM, Sanders VM (2007). Autonomic innervation and regulation of the immune system (1987-2007). Brain Behav Immun..

[15] Fliers E, Kreier F, Voshol PJ, Havekes LM, Sauerwein HP, Kalsbeek A (2003). White adipose tissue: getting nervous. J Neuroendocrinol..

[16] Ayala-Lopez N, Martini M, Jackson WF, Darios E, Burnett R, Seitz B (2014). Perivascular adipose tissue contains functional catecholamines. Pharmacol Res Perspect..

[17] Kasparov S, Teschemacher AG (2008). Altered central catecholaminergic transmission and cardiovascular disease. Exp Physiol..

[18] Szczepanska-Sadowska E, Cudnoch-Jedrzejewska A, Ufnal M, Zera T (2010). Brain and cardiovascular diseases: common neurogenic background of cardiovascular, metabolic and inflammatory diseases. J Physiol Pharmacol..

[19] McKinley MJ, Albiston AL, Allen AM, Mathai ML, May CN, McAllen RM (2003). The brain renin-angiotensin system: location and physiological roles. Int J Biochem Cell Biol..

[20] Ganong WF (2000). Circumventricular organs: definition and role in the regulation of endocrine and autonomic function. Clin Exp Pharmacol Physiol..

[21] Felder RB, Yu Y, Zhang ZH, Wei SG (2009). Pharmacological treatment for heart failure: a view from the brain. Clin Pharmacol Ther..

[22] Felder RB (2010). Mineralocorticoid receptors, inflammation and sympathetic drive in a rat model of systolic heart failure. Exp Physiol..

[23] Shi Z, Gan XB, Fan ZD, Zhang F, Zhou YB, Gao XY (2011). Inflammatory cytokines in paraventricular nucleus modulate sympathetic activity and cardiac sympathetic afferent reflex in rats. Acta Physiol..

[24] Saavedra JM, Angiotensin II (2012). AT(1) receptor blockers as treatments for inflammatory brain disorders. Clin Sci..

[25] Wu KL, Chan SH, Chan JY (2012). Neuroinflammation and oxidative stress in rostral ventrolateral medulla contribute to neurogenic hypertension induced by systemic inflammation. J Neuroinflammation.

[26] de Kloet AD, Krause EG, Shi PD, Zubcevic J, Raizada MK, Sumners C (2013). Neuroimmune communication in hypertension and obesity: a new therapeutic angle?. Pharmacol Ther..

[27] Paton JF, Wang S, Polson JW, Kasparov S (2008). Signalling across the blood brain barrier by angiotensin II: novel implications for neurogenic hypertension. J Mol Med..

[28] Zhang M, Mao Y, Ramirez SH, Tuma RF, Chabrashvili T (2010). Angiotensin II induced cerebral microvascular inflammation and increased blood-brain barrier permeability via oxidative stress. Neuroscience..

[29] Guillot FL, Audus KL (1991). Angiotensin peptide regulation of bovine brain microvessel endothelial cell monolayer permeability. J Cardiovasc Pharmacol..

[30] Fleegal-DeMotta MA, Doghu S, Banks WA (2009). Angiotensin II modulates BBB permeability via activation of the AT(1) receptor in brain endothelial cells. J Cereb Blood Flow Metab..

[31] Biancardi VC, Son SJ, Ahmadi S, Filosa JA, Stern JE (2014). Circulating angiotensin II gains access to the hypothalamus and brain stem during hypertension via breakdown of the blood-brain barrier. Hypertension..

[32] Labus J, Häckel S, Lucka L, Danker K (2014). Interleukin-1β induces an inflammatory response and the breakdown of the endothelial cell layer in an improved human THBMEC-based in vitro blood-brain barrier model. J Neurosci Methods..

[33] Rochfort KD, Collins LE, Murphy RP, Cummins PM (2014). Downregulation of blood-brain barrier phenotype by proinflammatory cytokines involves NADPH oxidase-dependent ROS generation: consequences for interendothelial adherens and tight junctions. PLoS One..

[34] Paton JF, Raizada MK (2010). Neurogenic hypertension. Exp Physiol..

[35] Reckelhoff JF, Romero JC (2003). Role of oxidative stress in angiotensin-induced hypertension. Am J Physiol Regul Integr Comp Physiol..

[36] Shi P, Diez-Freire C, Jun JY, Qi Y, Katovich MJ, Li Q (2010). Brain microglial cytokines in neurogenic hypertension. Hypertension..

[37] Kang YM, Ma Y, Zheng JP, Elks C, Sriramula S, Yang ZM (2009). Brain nuclear factor-kappa B activation contributes to neurohumoral excitation in angiotensin II-induced hypertension. Cardiovasc Res.

[38] Kang YM, He RL, Yang LM, Qin DN, Guggilam A, Elks C (2009). Brain tumour necrosis factor-alpha modulates neurotransmitters in hypothalamic paraventricular nucleus in heart failure. Cardiovasc Res..

[39] Cardinale JP, Sriramula S, Mariappan N, Agarwal D, Francis J (2012). Angiotensin II-induced hypertension is modulated by nuclear factor-kappa B in the paraventricular nucleus. Hypertension..

[40] Masson GS, Costa TS, Yshii L, Fernandes DC, Soares PP, Laurindo FR (2014). Time-dependent effects of training on cardiovascular control in spontaneously hypertensive rats: role for brain oxidative stress and inflammation and baroreflex sensitivity. PLoS One..

[41] Welch WJ, Chabrashvili T, Solis G, Chen Y, Gill PS, Aslam S (2006). Role of extracellular superoxide dismutase in the mouse angiotensin slow pressor response. Hypertension..

[42] Modlinger P, Chabrashvili T, Gill PS, Mendonca M, Harrison DG, Griendling KK (2006). RNA silencing in vivo reveals role of p22phox in rat angiotensin slow pressor response. Hypertension..

[43] Nguyen G, Muller DN (2010). The biology of the (pro)renin receptor. J Am Soc Nephrol..

[44] Zubcevic J, Jun JY, Lamont G, Murca TM, Shi P, Yuan W (2013). Nucleus of the solitary tract(pro)renin receptor-mediated antihypertensive effect involves nuclear factor kappa B-cytokine signaling in the spontaneously hypertensive rat. Hypertension..

[45] Shi P, Grobe JL, Desland FA, Zhou G, Shen XZ, Shan Z (2014). Direct pro-inflammatory effects of prorenin on microglia. PLoS One..

[46] Shan Z, Shi P, Cuadra AE, Dong Y, Lamont GJ, Li Q (2010). Involvement of the brain(pro)renin receptor in cardiovascular homeostasis. Circ Res..

[47] Okamoto K, Aoki K (1963). Development of a strain of spontaneously hypertensive rats. Jpn Circ J..

[48] Friese RS, Mahboubi P, Mahapatra NR, Mahata SK, Schork NJ, Schmid-Schönbein GW (2005). Common genetic mechanisms of blood pressure elevation in two independent rodent models of human essential hypertension. Am J Hypertens..

[49] Marvar PJ, Lob H, Vinh A, Zarreen F, Harrison DG (2011). The central nervous system and inflammation in hypertension. Curr Opin Pharmacol..

[50] Li W, Peng H, Cao T, Sato R, McDaniels SJ, Kobori H (2012). Brain-targeted (pro)renin receptor knockdown attenuates angiotensin II dependent hypertension. Hypertension..

[51] Li W, Peng H, Mehaffey EP, Kimball CD, Grobe JL, van Gool JM (2014). Ang II potentiates prorenin-induced TNFa production. Hypertension..

[52] Waki H, Gouraud SS, Maeda M, Raizada MK, Paton JF (2011). Contributions of vascular inflammation in the brainstem for neurogenic hypertension. Respir Physiol Neurobiol..

[53] Agarwal D, Welsch MA, Keller JN, Francis J (2011). Chronic exercise modulates RAS components and improves balance between pro- and anti-inflammatory cytokines in the brain of SHR. Basic Res Cardiol..

[54] Kumagai H, Oshima N, Matsuura T, Iigaya K, Imai M, Onimaru H (2012). Importance of rostral ventrolateral medulla neurons in determining efferent sympathetic nerve activity and blood pressure. Hypertens Res..

[55] Su Q, Qin DN, Wang FX, Ren J, Li HB, Zhang M (2014). Inhibition of reactive oxygen species in hypothalamic paraventricular nucleus attenuates the renin-angiotensin system and proinflammatory cytokines in hypertension. Toxicol Appl Pharmacol..

[56] Qin L, Liu Y, Wang T, Wei SJ, Block ML, Wilson B (2004). NADPH oxidase mediates lipopolysaccharide-induced neurotoxicity and proinflammatory gene expression in activated microglia. J Biol Chem..

[57] Qin L, Li G, Qian X, Liu Y, Wu X, Liu B (2005). Interactive role of the toll-like receptor4 and reactive oxygen species in LPS-induced microglia activation. Glia..

[58] Krishna M, Narang H (2008). The complexity of mitogen-activated protein kinases (MAPKs) made simple. Cell Mol Life Sci..

[59] Pawate S, Shen Q, Fan F, Bhat NR (2004). Redox regulation of glial inflammatory response to lipopolysaccharide and interferon gamma. J Neurosci Res..

[60] Zhang ZH, Yu Y, Wei SG, Felder RB (2010). Centrally administered lipopolysaccharide elicits sympathetic excitation via NAD(P)H oxidase-dependent mitogen-activated protein kinase signaling. J Hypertens..

[61] Zhang ZH, Wei SG, Francis J, Felder RB (2003). Cardiovascular and renal sympathetic activation by blood-borne TNF-alpha in rat: the role of central prostaglandins. Am J Physiol Regul Integr Comp Physiol..

[62] Gao L, Wang W, Li YL, Schultz HD, Liu D, Cornish KG (2004). Superoxide mediates sympathoexcitation in heart failure: roles of angiotensin II and NAD(P)H oxidase. Circ Res..

[63] Chan SH, Hsu KS, Huang CC, Wang LL, Ou CC, Chan JY (2005). NADPH oxidase-derived superoxide anion mediates angiotensin II-induced pressor effect via activation of p38 mitogen-activated protein kinase in the rostral ventrolateral medulla. Circ Res..

[64] Zucker IH, Gao L (2005). The regulation of sympathetic nerve activity by angiotensin II involves reactive oxygen species and MAPK. Circ Res..

[65] Wei SG, Yu Y, Zhang ZH, Weiss RM, Felder RB (2008). Mitogen-activated protein kinases mediate upregulation of hypothalamic angiotensin II type 1 receptors in heart failure rats. Hypertension..

[66] Wei SG, Yu Y, Zhang ZH, Weiss RM, Felder RB (2008). Angiotensin II-triggered p44/42 mitogen-activated protein kinase mediates sympathetic excitation in heart failure rats. Hypertension..

[67] Gao L, Wang W, Li YL, Schultz HD, Liu D, Cornish KG (2005). Sympathoexcitation by central ANG II: roles for AT1 receptor upregulation and NAD(P)H oxidase in RVLM. Am J Physiol Heart Circ Physiol..

[68] Lindley TE, Doobay MF, Sharma RV, Davisson RL (2004). Superoxide is involved in the central nervous system activation and sympathoexcitation of myocardial infarction-induced heart failure. Circ Res..

[69] Cani PD, Bibiloni R, Knauf C, Waget A, Neyrinck AM, Delzenne NM (2008). Changes in gut microbiota control metabolic endotoxemia-induced inflammation in high-fat diet-induced obesity and diabetes in mice. Diabetes..

[70] Terra X, Montagut G, Bustos M, Llopiz N, Ardèvol A, Bladé C (2009). Grape-seed procyanidins prevent low-grade inflammation by modulating cytokine expression in rats fed a high-fat diet. J Nutr Biochem..

[71] Endo Y, Tomofuji T, Ekuni D, Irie K, Azuma T, Tamaki N (2010). Experimental periodontitis induces gene expression of proinflammatory cytokines in liver and white adipose tissue in obesity. J Periodontol..

[72] Manco M, Putignani L, Bottazzo GF (2010). Gut microbiota, lipopolysaccharides, and innate immunity in the pathogenesis of obesity and cardiovascular risk. Endocr Rev..

[73] Marvar PJ, Thabet SR, Buzik TJ, Lob HE, McCann LA, Weyand C (2010). Central and peripheral mechanisms of T-lymphocyte activation and vascular inflammation produced by angiotensin II-induced hypertension. Circ Res..

[74] Carrasquillo Y, Burkhalter A, Nerbonne JM (2012). A-type K+ channels encoded by Kv4.2, Kv4.3 and Kv1.4 differentially regulate intrinsic excitability of cortical pyramidal neurons. J Physiol.

[75] Xu H, Barnes GT, Yang Q, Tan G, Yang D, Chou CJ (2003). Chronic inflammation in fat plays a crucial role in the development of obesity-related insulin resistance. J Clin Invest..

[76] Thaler JP, Yi CX, Schur EA, Guyenet SJ, Hwang BH, Dietrich MO (2012). Obesity is associated with hypothalamic injury in rodents and humans. J Clin Invest..

[77] Yi CX, Al-Massadi O, Donelan E, Lehti M, Weber J, Ress C (2012). Exercise protects against high-fat diet-induced hypothalamic inflammation. Physiol Behav..

[78] Buckman LB, Thompson MM, Moreno HN, Ellacott KL (2013). Regional astrogliosis in the mouse hypothalamus in response to obesity. J Comp Neurol..

[79] Hsuchou H, He Y, Kastin AJ, Tu H, Markadakis EN, Rogers RC (2009). Obesity induces functional astrocytic leptin receptors in hypothalamus. Brain..

[80] de Kloet AD, Pioquinto DJ, Nguyen D, Wang L, Smith JA, Hiller H (2014). Obesity induces neuroinflammation mediated by altered expression of the renin-angiotensin system in mouse forebrain nuclei. Physiol Behav..

[81] Hilzendeger AM, Morgan DA, Brooks L, Dellsperger D, Liu X, Grobe JL (2012). A brain leptin-renin angiotensin system interaction in the regulation of sympathetic nerve activity. Am J Physiol Heart Circ Physiol..

[82] Arnold AC, Shaltout HA, Gallagher PE, Diz DI (2009). Leptin impairs cardiovagal baroreflex function at the level of the solitary tract nucleus. Hypertension..

[83] Hayes MR, Skibicka KP, Leichner TM, Guarnieri DJ, DiLeone RJ, Bence KK (2010). Endogenous leptin signaling in the caudal nucleus tractus solitarius and area postrema is required for energy balance regulation. Cell Metab..

[84] Mark AL, Agassandian K, Morgan DA, Liu X, Cassell MD, Rahmouni K (2009). Leptin signaling in the nucleus tractus solitarii increases sympathetic nerve activity to the kidney. Hypertension..

[85] Smith PM, Chambers AP, Price CJ, Ho W, Hopf C, Sharkey KA (2009). The subfornical organ: a central nervous system site for actions of circulating leptin. Am J Physiol Regul Integr Comp Physiol..

[86] Smith PM, Ferguson AV (2012). Cardiovascular actions of leptin in the subfornical organ are abolished by diet-induced obesity. J Neuroendocrinol..

[87] Marsh AJ, Fontes MA, Killinger S, Pawlak DB, Polson JW, Dampney RA (2003). Cardiovascular responses evoked by leptin acting on neurons in the ventromedial and dorsomedial hypothalamus. Hypertension..

[88] Akiyama H, Ikeda K, Katoh M, McGeer EG, McGeer PL (1994). Expression of MRP14, 27E10, interferon-alpha and leukocyte common antigen by reactive microglia in postmortem human brain tissue. J Neuroimmunol..

[89] Sedgwick JD, Ford AL, Foulcher E, Airriess R (1998). Central nervous system microglial cell activation and proliferation follows direct interaction with tissue-infiltrating T cell blasts. J Immunol..

[90] Nikodemova M, Watters JJ (2012). Efficient isolation of live microglia with preserved phenotypes from adult mouse brain. J Neuroinflammation..

[91] Weisberg SP, McCann D, Desai M, Rosenbaum M, Leibel RL, Ferrante AW (2003). Obesity is associated with macrophage accumulation in adipose tissue. J Clin Invest..

[92] Buckman LB, Hasty AH, Flaherty DK, Buckman CT, Thompson MM, Matlock BK (2014). Obesity induced by a high-fat diet is associated with increased immune cell entry into the central nervous system. Brain Behav Immun..

[93] Simard AR, Soulet D, Gowing G, Julien JP, Rivest S (2006). Bone marrow-derived microglia play a critical role in restricting senile plaque formation in Alzheimer’s disease. Neuron..

